# Gender Differences in COVID-19 Lockdown Impact on Mental Health of Undergraduate Students

**DOI:** 10.3389/fpsyt.2021.813130

**Published:** 2022-01-05

**Authors:** Andrea Amerio, Paola Bertuccio, Francesca Santi, Davide Bianchi, Andrea Brambilla, Alessandro Morganti, Anna Odone, Alessandra Costanza, Carlo Signorelli, Andrea Aguglia, Gianluca Serafini, Stefano Capolongo, Mario Amore

**Affiliations:** ^1^Department of Neuroscience, Rehabilitation, Ophthalmology, Genetics, Maternal and Child Health (DINOGMI), Section of Psychiatry, University of Genoa, Genoa, Italy; ^2^Istituto di Ricovero e Cura a Carattere Scientifico (IRCCS) Ospedale Policlinico San Martino, Genoa, Italy; ^3^Department of Public Health, Experimental and Forensic Medicine, University of Pavia, Pavia, Italy; ^4^Politecnico di Milano, Department of Architecture, Built Environment and Construction Engineering, Design and Health Lab, Milan, Italy; ^5^Department of Psychiatry, Faculty of Medicine, University of Geneva (UNIGE), Geneva, Switzerland; ^6^School of Medicine, Vita-Salute San Raffaele University, Milan, Italy

**Keywords:** COVID-19, undergraduate student, mental health, gender, lockdown

## Abstract

**Background:** Prolonged university closures and social distancing-imposed measures due to the COVID-19 pandemic obliged students to at-home learning with online lectures and educational programs promoting potential social isolation, loneliness, hopelessness, and episodes of clinical decompensation.

**Methods:** A web-based cross-sectional survey was carried out in a university institute in Milan, Northern Italy, to assess the COVID-19 lockdown impact on the mental health of the undergraduate students. We estimated the odds ratios (OR) and the corresponding 95% confidence intervals (CI) using adjusted logistic regression models.

**Results:** Of the 8,177 students, 12.8% reported depressive symptoms, 25.6% anxiety, 8.7% insomnia, and 10.6% reported impulsive tracts, with higher proportions among females than males. Mental health symptoms were positively associated with caring for a person at home, a poor housing quality, and a worsening in working performance. Among males compared with females, a poor housing quality showed a stronger positive association with depressive symptoms and impulsivity, and a worsening in the working performance was positively associated with depressive and anxiety symptoms. In addition, the absence of private space was positively associated with depression and anxiety, stronger among males than females.

**Conclusions:** To our knowledge, this is the first multidisciplinary consortium study, involving public mental health, environmental health, and architectural design. Further studies are needed to confirm or refute our findings and consequent recommendations to implement well-being interventions in pandemic conditions.

## Introduction

Italy holds one of the highest COVID-19 clinical burden worldwide and the Lombardy region—one of the richest and most productive area in the whole of Europe—was among the first hit in Europe and, within Italy, accounts for >50% of all COVID-19 deaths (1). Lockdown measures have been adopted by the Italian government in order to help curb the pandemic, including, by March 5, school and university closures (2). The life of millions of Italians suddenly changed, and lifestyle habits have been substantially modified (3), with anticipated short-term consequences on physical and mental health (4, 5).

University education is a crucial period in a transitional age, between adolescence and adulthood, because of the higher distress that students are exposed to compared with the general population (6). Emancipation, financial self-sufficiency, career choices, and intimate and friendship relationships are just some of the challenges that undergraduate students are faced with.

These years coincide with the peak period of risk for the onset of mental disorders since ~75% of all lifetime mental disorders have their onset prior to the age of 24 (7). In particular, mental disorders during this period can be associated with negative effects on the development of young people, including worsening academic performance, dropout from university, and long-term negative impact on later adult labor market functioning, relationship functioning, and health (8, 9).

Psychological response following exposure to stressful events is extremely heterogeneous. People can show a high degree of resilience and quickly return to normal lives or develop different kinds and degrees of psychiatric symptoms. Males and females present different reactions to stress, different ways to manage stress, and to perceive their ability to do so. Findings from the literature suggest that while females are more likely to report physical symptoms associated with stress, they better connect with others in their lives, and at times, these connections are important to their stress management strategies (10).

In the COVID-19 lockdown context, prolonged university closures and social distancing-imposed measures obliged students to at-home learning with online lectures and educational programs promoting potential social isolation, loneliness, hopelessness, and episodes of clinical decompensation (11). For over 2 months of stay-at-home orders, houses became the only place where students slept, ate, studied, practiced sports, and socialized.

To the best of our knowledge, original studies investigating mental health consequences of COVID-19 lockdown on Italian undergraduate students are still scant and conducted on small samples (12, 13). We aimed to evaluate the impact of COVID-19 mandatory confinement on the mental health of the Italian undergraduate student population with particular regard to gender differences and housing quality.

## Methods

### Survey Sample

We used data from a large web-based cross-sectional survey conducted in the Lombardy region to assess the mental health impact of the first wave of COVID-19 mass quarantine restrictions. Details were described elsewhere (14). In brief, a web-based survey questionnaire was sent by mail from April 1, 2020 to May 1, 2020 to all personnel from Politecnico di Milano, a scientific–technological university institute in Milan, Lombardy Region, Italy. The total sample (*N* = 9,261) consisted of undergraduate students, PhD students, teaching staff, and administrative personnel, aged ≥ 18 years old. The survey was anonymous, and confidentiality of information was assured. A written consent was given to all individuals before participating in the questionnaire/study. Participants were allowed to terminate the survey at any time they desired and no monetary rewards were given for completing the questionnaire.

We restricted our study on the subsample of 8,177 students, to avoid recruitment bias and yield a homogeneous group, separately among males (*n* = 4,095) and females (*n* = 4,082).

### Questionnaire

The questionnaire, filled in through a free Google Forms platform, consisted of three main sections. The first one investigated general characteristics of participants, including gender, current age, marital status, education level, and subjective impact of the mandatory confinement on working performance. The second section consisted of the administration of some evaluation scales of the mental health status, designed to recognize depressive and anxiety symptoms, insomnia, and impulsivity traits. The third section investigated the physical and architectural housing characteristics.

### Study Outcomes

We derived the outcomes of the study from four evaluation scales designed to recognize depressive and anxiety symptoms, insomnia, and impulsivity traits. We used the following cutoffs to obtain binary outcomes:

1. For the nine-item Patient Health Questionnaire (PHQ-9) (15), we considered the cutoff for depressive symptoms at ≥15 (moderate and severe depressive symptoms);
2. For the seven-item Generalized Anxiety Disorder (GAD-7) (16), we considered the cutoff for anxiety symptoms at ≥10 (moderate and severe anxiety symptoms);
3. For the seven-item Insomnia Severity Index (ISI) (17), we considered the cutoff for insomnia at ≥15 (moderate and severe);
4. For the Barratt Impulsiveness Scale-11 (BIS-11) (18), we considered the cutoff for trait impulsivity at ≥70, and below or above the highest quartile for the three impulsivity components (i.e., attentional, motor, and non-planning).

### Exposure Factors

In line with previous studies in the field of Environmental Psychology and Evidence-Based Design (19, 20), we considered as possible associated factors to mental health some selected physical and architectural housing characteristics, including the apartment dimension (in terms of net square meters), the presence/absence of a livable outdoor space (balcony or garden), the view typology (green or buildings), and a score to define the quality of the indoor space. The score was obtained by a set of seven parameters: natural lighting, acoustic comfort, thermohygrometric comfort, need for artificial lighting during the day, presence/absence of soft qualities in the living area, such as art objects or greenery/plants, and presence/absence of privacy. Then, we considered three categories of the quality of the indoor area as high (6–7 satisfied parameters), medium (4–5 satisfied parameters), or poor (0–3 satisfied parameters). Finally, we considered as potential factors associated to mental outcomes as caring for a person at home during the confinement and the subjective impact of the mandatory confinement in terms of worsening in working performance.

### Statistical Analysis

The analyses were conducted separately by sex. Proportions of the mental health outcomes between males and females were compared using the chi-square test. As the main analysis, we estimated odds ratios (OR) of reporting mental health symptoms, and the corresponding 95% confidence intervals (CI), using adjusted logistic regression models. The models included age and the variables that showed a *p*-value < 0.25 in the multivariable models as dependent variables, i.e., caring for a person at home (no/yes), apartment dimension (>100 mq, 81–100 mq, and <80 mq), the quality indoor score (high, medium, and poor), and a worsening in working performance (none/little and much/very much) (21).

As a secondary analysis, we estimated the associations between each outcome and selected components of the indoor quality score, in order to explore which of them were more linked to mental health symptoms.

We verified the heterogeneity among strata of sex using the Cochran's *Q* test statistic (22). We carried out the aforementioned statistical analyses with the SAS software, version 9.4 (SAS Institute, Cary, NC, USA), and the software R version 3.4.1 (R Foundation for Statistical Computing, Vienna, Austria).

## Results

A total of 8,177 students completed the survey, and the overall response rate (ORR) was around 31.5%. Of the 8,177 students, 12.8% reported depressive symptoms, 25.6% anxiety, 8.7% insomnia, and 10.6% reported impulsive tracts. These proportions were higher among the females than among males, with 15.4% of females reporting depressive symptoms, 33% anxiety, 9.5% insomnia, and 11.4% impulsivity (Table 1). Considering the three impulsivity components, the attentional one was more frequently reported among females, while the motor one was more frequently reported among males. Non-planning impulsivity was similarly reported among males and females.

**Table 1:** Distribution of 8,177 students according to the mental health outcomes and sex.

	**Total**		**Males**		**Females**		*p*-value^*^
	***N*** **=** **8,177**		***N*** **=** **4,095**		***N*** **=** **4,082**		
	***N***	**%**	***N***	**%**	***N***	**%**	
**Patient health questionnaire (PHQ-9)**							
<15	7,127	87.2	3,674	89.7	3,453	84.6	<0.001
≥15	1,050	12.8	421	10.3	629	15.4	
**General anxiety disorder (GAD-7)**							
<10	6,080	74.4	3,345	81.7	2,735	67.0	<0.001
≥10	2,097	25.6	750	18.3	1,347	33.0	
**Insomnia severity index (ISI)**							
<15	7,466	91.3	3,773	92.1	3,693	90.5	0.01
≥15	711	8.7	322	7.9	389	9.5	
**Barratt impulsiveness scale (BIS-11)**							
<70	7,307	89.4	3,691	90.1	3,616	88.6	0.02
≥70	870	10.6	404	9.9	466	11.4	
**Barratt impulsiveness scale: attentional**							
< I quartile (8–12)	1,229	15.0	670	16.4	559	13.7	
I–III quartile (13–17)	4,598	56.2	2,333	57.0	2,265	55.5	<0.001
>III quartile (18–30)	2,350	28.7	1,092	26.7	1,258	30.8	
**Barratt impulsiveness scale: motor**							
< I quartile (11–16)	1,777	21.7	798	19.5	979	24.0	
I–III quartile (17–20)	3,642	44.5	1,853	45.3	1,789	43.8	<0.001
>III quartile (21–36)	2,758	33.7	1,444	35.3	1,314	32.2	
**Barratt impulsiveness scale: non-planning**							
< I quartile (11–19)	1,553	19.0	808	19.7	745	18.3	
I–III quartile (20–25)	4,005	49.0	2,015	49.2	1,990	48.8	0.09
>III quartile (26–40)	2,619	32.0	1,272	31.1	1,347	33.0	

* *Chi-squared test (p ≤ 0.05 identifies statistically significant differences between males and females)*.

Table 2 shows the associations between selected exposures and mental health symptoms. Caring for a person at home was positively associated to all the studied outcomes among both sexes, except for impulsivity among females. In addition, caring for a person at home had a stronger association to insomnia among males (OR 2.12, 95% CI: 1.59–2.83) compared with females (OR 1.19, 95% CI: 0.90–1.58). Similarly, a poor housing quality was positively associated to all symptoms, with stronger associations among males compared with females for depressive symptoms (OR 4.75, 95% CI: 3.44–6.57 vs. 2.62, 95% CI: 2.04–3.37) and impulsivity (OR 2.25, 95% CI: 1.67–3.03 vs. 1.40, 95% CI: 1.06–1.86). Finally, a worsening in working performance showed positive associations with all symptoms among both sexes, with stronger associations among males than females for depressive and anxiety symptoms. Table 3 shows the association between the quality indoor score and the three impulsivity components. Compared with high-quality score, a poor-quality indoor score was positively associated to attentional and non-planning impulsivity traits, similarly in both sexes (Table 3). These associations were higher among the males than females (with a significant difference for the medium-quality score). No associations emerged with the motor impulsivity.

**Table 2:** Odds ratio^*^ (OR) and their 95% confidence intervals (CI) for symptoms of depression [nine-item Patient Health Questionnaire (PHQ-9) ≥ 15], anxiety [seven-item Generalized Anxiety Disorder (GAD-7) ≥ 10], insomnia [Insomnia Severity Index (ISI) ≥ 15], and impulsivity [Barratt Impulsiveness Scale-11 (BIS-11) ≥ 70] according to selected factors, in males and females, separately.

	**Total**	**PHQ-9 (≥15 vs**. ** <15)**		**GAD-7 (≥10 vs**. ** <10)**		**ISI (≥15 vs**. ** <15)**		**BIS-11 (≥70 vs**. ** <70)**	
	***N***	**Males**	**Females**	**Males**	**Females**	**Males**	**Females**	**Males**	**Females**
**Caring for a person at home**									
No	6,986	1	1	1	1	1	1	1	1
Yes	1,191	1.75 (1.32–2.31)	1.28 (1.01–1.63)	1.43 (1.14–1.79)	1.49 (1.24–1.79)	**2.12 (1.59–2.83)**	**1.19 (0.90–1.58)**	1.38 (1.04–1.82)	0.96 (0.73–1.26)
**Apartment dimension (mq)**									
>100	4,860	1	1	1	1	1	1	1	1
81–100	1,787	1.09 (0.83–1.43)	0.86 (0.68–1.08)	1.08 (0.88–1.33)	0.89 (0.75–1.07)	1.30 (0.98–1.73)	0.76 (0.57–1.02)	1.07 (0.83–1.39)	0.84 (0.64–1.09)
≤80	1,530	1.31 (1.00–1.73)	1.23 (0.98–1.55)	1.18 (0.94–1.47)	1.17 (0.98–1.41)	1.16 (0.85–1.58)	1.05 (0.8–1.38)	1.02 (0.77–1.35)	1.27 (0.99–1.63)
**Balcony livable**									
Yes	5,964	1	1	1	1	1	1	1	1
No	2,213	1.05 (0.83–1.33)	1.24 (1.02–1.52)	0.94 (0.78–1.14)	1.15 (0.98–1.35)	1.09 (0.84–1.41)	1.26 (0.99–1.6)	1.33 (1.06–1.68)	1.08 (0.86–1.35)
**View from apartment**									
Green	3,304	1	1	1	1	1	1	1	1
Buildings	4,873	1.16 (0.92–1.46)	1.03 (0.85–1.24)	0.97 (0.82–1.16)	0.90 (0.78–1.04)	1.25 (0.97–1.61)	1.01 (0.81–1.26)	1.07 (0.86–1.33)	1.01 (0.82–1.24)
**Quality indoor score**									
High	3,335	1	1	1	1	1	1	1	1
Medium	3,560	**2.09 (1.56–2.80)**	**1.37 (1.11–1.70)**	1.52 (1.24–1.86)	1.45 (1.24–1.69)	1.36 (1.01–1.83)	1.57 (1.22–2.02)	1.18 (0.92–1.51)	0.99 (0.79–1.23)
Poor	1,282	**4.75 (3.44–6.57)**	**2.62 (2.04–3.37)**	3.08 (2.42–3.93)	2.25 (1.83–2.76)	3.05 (2.19–4.26)	2.01 (1.47–2.75)	**2.25 (1.67–3.03)**	**1.40 (1.06–1.86)**
**Worsening in working performance**									
None/little	5,532	1	1	1	1	1	1	1	1
Much/very much	2,645	**5.57 (4.42–7.01)**	**3.53 (2.94–4.24)**	**3.06 (2.58–3.62)**	**2.29 (1.98–2.63)**	2.92 (2.29–3.71)	2.11 (1.7–2.63)	1.93 (1.56–2.39)	1.72 (1.40–2.10)

* *Estimates obtained from multivariable logistic regression models, adjusted for age at interview, caring for a person at home, apartment dimension (mq), worsening in working performance, and quality indoor score*.

*Significant differences are highlighted in bold*.

**Table 3:** Odds ratio^*^ (OR) and their 95% confidence intervals (CI) for the association between the quality indoor score and the three impulsivity components, in males and females, separately.

	**BIS: attentional**		**BIS: motor**		**BIS: non-planning**	
	**Males**	**Females**	**Males**	**Females**	**Males**	**Females**
**Quality indoor score (ref: high)**						
Medium	1.18 (1.01–1.39)	1.06 (0.91–1.23)	1.05 (0.91–1.21)	0.87 (0.75–1.01)	**1.33 (1.14–1.54)**	**1.05 (0.90–1.21)**
Poor	1.77 (1.43–2.20)	1.46 (1.19–1.80)	1.05 (0.86–1.29)	1.14 (0.93–1.39)	1.61 (1.31–1.98)	1.37 (1.12–1.67)

* *Estimates obtained from multivariable logistic regression models, adjusted for age at interview, caring for a person at home, apartment dimension (mq), and worsening in working performance. Reference category: 1*.

*Significant differences are highlighted in bold*.

**Figure 1:**
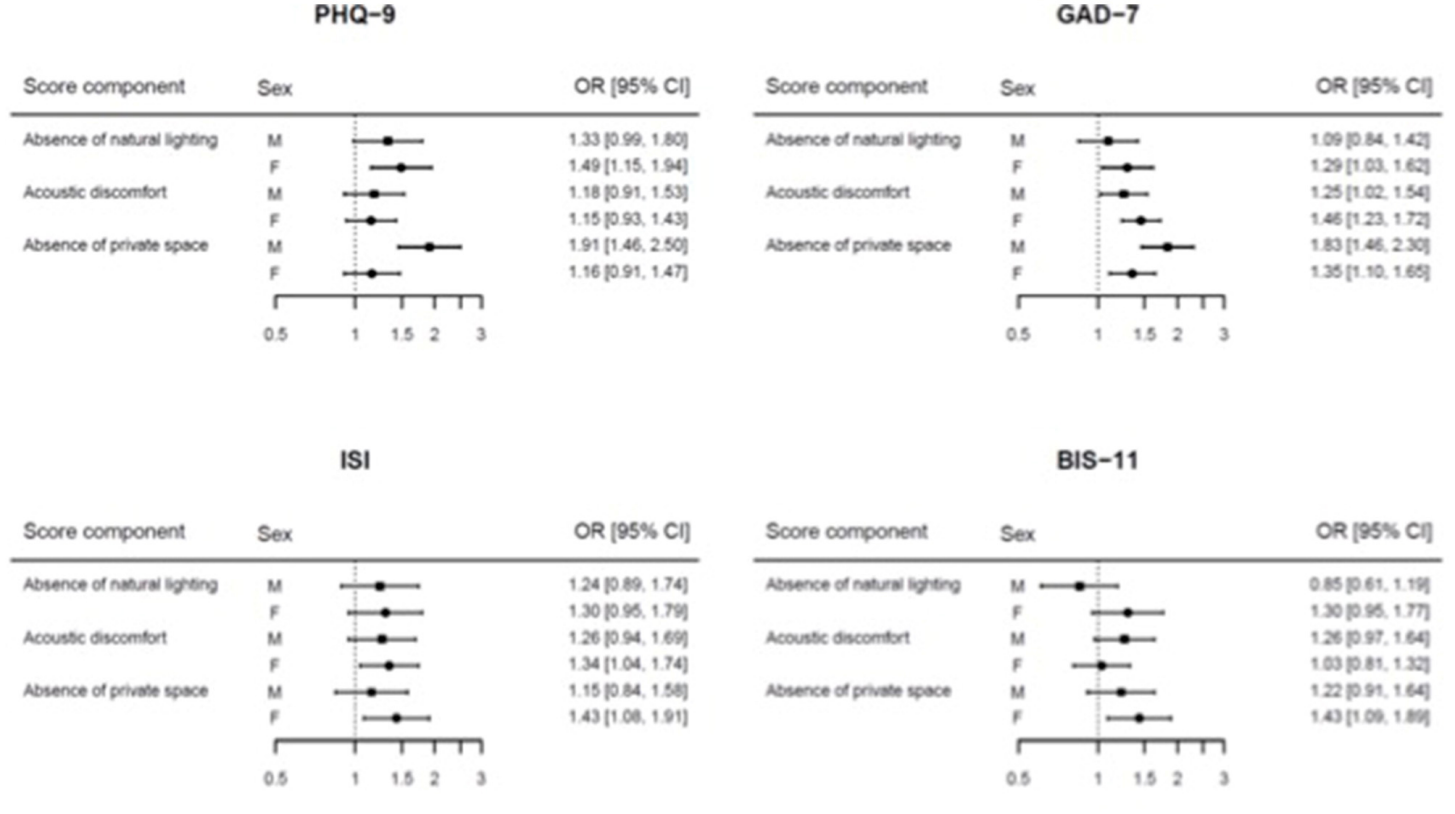
Forest plot of the odds ratio (OR) and their 95% confidence intervals (CI) for the association between three selected components of the quality indoor score and the four mental health outcomes.

Figure 1 shows the associations between three selected components of the indoor quality score (i.e., absence of natural lighting, acoustic discomfort, and absence of private space) and the four mental health outcomes. The absence of a private space at home was the architectural parameter mainly associated to mental health symptoms, with the strongest positive associations among males for depressive symptoms (OR 1.91, 95% CI: 1.46–2.50) and anxiety (OR 1.83, 95% CI: 1.46–2.30). Among females, the strongest positive associations emerged with insomnia (OR 1.43, 95% CI: 1.08–1.91) and impulsivity (OR 1.43, 95% CI: 1.09–1.89). The differences between males and females were statistically significant for the PHQ-9 outcome (*p* for heterogeneity = 0.007) and borderline for the GAD-7 (*p* = 0.051). Finally, the absence of natural lighting and acoustic discomfort showed positive associations with the studied outcomes, with the strongest ones between the absence of natural lighting and depression, and between acoustic discomfort and anxiety among females. However, these associations were not statistically different with those among males.

## Discussion

The mental health impact of the COVID-19 mandatory confinement on undergraduate Italian students was worst among females than males. Mental health symptoms were positively associated to caring for a person at home, living in a poor housing quality, and a worsening in working performance. Male students who were caring for a person at home during the confinement reported more frequent insomnia than females. Similarly, males who lived in a poor housing quality reported more frequent depressive symptoms and impulsivity than females, as well as males who declared a worsening in working performance reported more frequent depressive and anxiety symptoms than females. In addition, a poor housing quality was associated also to attentional and non-planning impulsivity, and males who lived in the absence of a private space reported more frequently depression and anxiety than females.

Our findings should be interpreted in light of the pervasive impact that the national lockdown adopted to contain the spread of the infection may have had on physical and mental health.

The result that females compared with males, among undergraduate students, worsened their mental health status more, is in line with current national and international COVID-19 literature that estimates a greater risk in females than males in developing depression, anxiety, paranoid ideations, post-traumatic stress symptoms, sleep disorders, and a worsening in the interpersonal sensitivity dimension (13, 23–26).

Caring for a person at home can be a source of emotional distress, especially for the youth (27). As reported by our findings, in the time of the COVID-19 lockdown restrictions, being forced to stay together and sharing the same living space for many weeks/months resulted in a worsening of the burden of the caregiver with a higher prevalence of sleep disturbance in males than in females.

Depressive and anxiety symptoms were more likely observed in undergraduate males who reported a worsening in working performance. Compared with females, the reduced ability of males to cope with adversity and to tolerate uncertainty without knowing what their future will be (28) could explain the difficulties to plan effective study sessions and keep the concentration on online university courses with mental health consequences.

Findings from our survey reported a strong positive association between poor housing quality and mental health outcomes such as depressive symptoms and impulsivity, mainly observed in undergraduate males, with particular regard to attentional and non-planning impulsivity. The association observed between a poor-quality indoor space that do not guarantee adequate privacy and a worsening in depressive symptoms and impulsivity can be interpreted both in light of higher fear of infection as well as a proxy for lower socioeconomic status with consequent higher uncertainty about the future associated to household-level economic impact of the COVID-19 response (29).

As confirmed by recent evidence from the literature, impulsivity traits, male gender, and young age are considered risk factors for gambling onset especially among low socioeconomic status youth (30, 31). In the COVID-19 era, this is even more important because the development of technology, which facilitates the possibility of gambling from home and the period of isolation caused by the COVID-19 pandemic resulted in more opportunities to gamble online (32).

This study needs to be interpreted in the light of several strengths and limitations. Among its strengths, the large homogeneous sample size and the use of validated evidence-based psychiatric assessment tools. Among its limitations, the use of self-reporting questionnaires, the cross-sectional study design, the low response rate, and the enrollment of students from a single university that limited the generalizability of the results. In particular, the cross-sectional design study does not allow inferences on the temporal relationship between the variables and only shows measures of associations. Moreover, no information on the mental health status of the participants before the COVID-19 outbreak are available. Last, housing physical characteristics have been investigated with an *ad-hoc* not questionnaire due to the scant evidence published in the literature.

In the first weeks of the pandemic, March 2020, a panel of experts convened by the UK Academy of Medical Sciences and a mental health research charity (MQ: Transforming Mental Health) set out immediate priorities and longer-term strategies for research and encouraged the collection of high-quality data on the COVID-19 impact on mental health across the whole population, with particular regard to vulnerable populations including the youth, through the integration across different disciplines and sectors (33). To our knowledge, this is the first multidisciplinary consortium study, involving public mental health, environmental health, and architectural design, conducted on a large sample of undergraduate students in Lombardy, the Italian region most affected by the pandemic, exploring the effects of the COVID-19 lockdown restrictions on a rich set of mental health outcomes.

University years coincide with the peak period of risk for the onset of mental disorders (34), and they are associated with a significant increase in risky health behaviors (35). Considering the importance of undergraduate students to the future social capital of society and the potential negative impact of mental health problems on their lives, prevention and early treatment of mental health problems in these specific years represent a key public health priority (36–38).

Results from our study confirm a built environment as a key determinant of health, whose quality builds on the availability of resources, site location planning, and green spaces. An interdisciplinary approach involving urban planning, public mental health, environmental health, epidemiology, and sociology, is needed to inform welfare and housing policies centered on population well-being, especially in the COVID-19 times.

Further studies are needed to confirm or refute our findings and consequent recommendations to implement well-being interventions in pandemic conditions. A careful and comprehensive analysis of risk and protective factors in the individual and environmental context should be performed in order to early detect peculiar needs of care as well as plan and implement appropriate and targeted interventions centered on vulnerable population health (39).

## Data Availability Statement

The raw data supporting the conclusions of this article will be made available by the authors, without undue reservation.

## Ethics Statement

Approval was given by IRCCS Ospedale Policlinico San Martino of Genoa. The patients/participants provided their written informed consent to participate in this study.

## Author Contributions

AAm, AM, and SC conceptualized the study. FS and DB curated the data. PB performed the formal analysis. AB and AC conducted the investigation. AAg and GS formulated the methodology. SC and MA were in charge of the project administration. CS, SC, and MA supervised the study. AAm and AO wrote the original draft. CS, GS, SC, and MA reviewed and edited the manuscript. All authors have read and agreed to the present version of the manuscript.

## Conflict of Interest

The authors declare that the research was conducted in the absence of any commercial or financial relationships that could be construed as a potential conflict of interest.

## Publisher's Note

All claims expressed in this article are solely those of the authors and do not necessarily represent those of their affiliated organizations, or those of the publisher, the editors and the reviewers. Any product that may be evaluated in this article, or claim that may be made by its manufacturer, is not guaranteed or endorsed by the publisher.

## References

[1] Signorelli C Scognamiglio T Odone A. COVID-19 in Italy: impact of containment measures and prevalence estimates of infection in the general population. Acta Biomed. (2020) 91:175–9. 10.23750/abm.v91i3-S.9511 32275287 PMC7975916

[2] Odone A Delmonte D Scognamiglio T Signorelli C. COVID-19 deaths in Lombardy, Italy: data in context. Lancet Public Health. (2020) 5:e310. 10.1016/S2468-2667(20)30099-2 32339478 PMC7182509

[3] Odone A Lugo A Amerio A Borroni E Bosetti C Carreras G. COVID-19 lockdown impact on lifestyle habits of Italian adults. Acta Biomed. (2020) 91:87–9. 10.23750/abm.v91i9-S.10122 32701921 PMC8023096

[4] Costanza A Mazzola V Radomska M Amerio A Aguglia A Prada P. Who consult an adult psychiatric emergency department? Pertinence of admissions and opportunities for telepsychiatry. Medicina. (2020) 56:295. 10.3390/medicina56060295 32545811 PMC7353920

[5] Amerio A Lugo A Stival C Fanucchi T Gorini G Pacifici R. COVID-19 lockdown impact on mental health in a large representative sample of Italian adults. J Affect Disord. (2021) 292:398–404. 10.1016/j.jad.2021.05.117 34139414 PMC8777065

[6] Arnett JJ Žukauskiene R Sugimura K. The new life stage of emerging adulthood at ages 18-29 years: implications for mental health. Lancet Psychiatry. (2014) 1:569–76. 10.1016/S2215-0366(14)00080-7 26361316

[7] Kessler RC Berglund P Demler O Jin R Merikangas KR Walters EE. Lifetime prevalence and age-of-onset distributions of DSM-IV disorders in the national comorbidity survey replication. Arch Gen Psychiatry. (2005) 62:5939–602. 10.1001/archpsyc.62.6.593 15939837

[8] Scott KM Lim C Al-Hamzawi A Alonso J Bruffaerts R Caldas-de-Almeida JM. Association of mental disorders with subsequent chronic physical conditions: world mental health surveys from 17 countries. JAMA Psychiatr. (2016) 73:150–8. 10.1001/jamapsychiatry.2015.2688 26719969 PMC5333921

[9] Bruffaerts R Mortier P Kiekens G Auerbach RP Cuijpers P Demyttenaere K. Mental health problems in college freshmen: prevalence and academic functioning. J Affect Disord. (2018) 225:97–103. 10.1016/j.jad.2017.07.044 28802728 PMC5846318

[10] Goldfarb EV Seo D Sinha R. Sex differences in neural stress responses and correlation with subjective stress and stress regulation. Neurobiol Stress. (2019) 11:100177. 10.1016/j.ynstr.2019.100177 31304198 PMC6603439

[11] Tang W Hu T Hu B Jin C Wang G Xie C. Prevalence and correlates of PTSD and depressive symptoms one month after the outbreak of the COVID-19 epidemic in a sample of home-quarantined Chinese university students. J Affect Disord. (2020) 274:1–7. 10.1016/j.jad.2020.05.009 32405111 PMC7217769

[12] Nania T Dellafiore F Caruso R Barello S. Risk and protective factors for psychological distress among Italian university students during the COVID-19 pandemic: The beneficial role of health engagement. Int J Soc Psychiatry. (2021) 67:102–3. 10.1177/0020764020945729 32721256

[13] Marelli S Castelnuovo A Somma A Castronovo V Mombelli S Bottoni D. Impact of COVID-19 lockdown on sleep quality in university students and administration staff. J Neurol. (2021) 268:8–15. 10.1007/s00415-020-10056-6 32654065 PMC7353829

[14] Amerio A Brambilla A Morganti A Aguglia A Bianchi D Santi F. COVID-19 lockdown: housing built environment's effects on mental health. Int J Environ Res Public Health. (2020) 17:5973. 10.3390/ijerph17165973 32824594 PMC7459481

[15] Spitzer R Kroken K Williams JB. Validation and utility of a self-report version of PRIME-MD: The PHQ primary care study. Primary care evaluation of mental disorders Patient health questionnaire. JAMA. (1999) 282:1737. 10.1001/jama.282.18.1737 10568646

[16] Spitzer RL Kroenke K Williams JBW Lowe B. A brief measure for assessing generalized anxiety disorder: The GAD-7. Arch Intern Med. (2006) 166:1092. 10.1001/archinte.166.10.1092 16717171

[17] Morin CM Belleville G Bélanger L Ivers H. The insomnia severity index: psychometric indicators to detect insomnia cases and evaluate treatment response. Sleep. (2011) 34:601–8. 10.1093/sleep/34.5.601 21532953 PMC3079939

[18] Patton JH Stanford MS Barratt ES. Factor structure of the Barratt impulsiveness scale. J Clin Psychol. (1995) 51:768–74. 10.1002/1097-4679(199511)51:6<768::AID-JCLP2270510607>3.0.CO;2-1 8778124

[19] Brambilla A Rebecchi A Capolongo S. Evidence based hospital design. A literature review of the recent publications about the EBD impact of built environment on hospital occupants' and organizational outcomes. Ann Ig. (2019) 31:165–80. 10.7416/ai.2019.2269 30714614

[20] Capolongo S Buffoli M Brambilla A Rebecchi A. Healthy urban planning & design strategies to improve urban quality and attractiveness of places. TECHNE J Technol Architect Environ. (2020) 19:271–9.

[21] Bursac Z Gauss CH Williams DK Hosmer DW. Purposeful selection of variables in logistic regression. Source Code Biol Med. (2008) 3:17. 10.1186/1751-0473-3-17 19087314 PMC2633005

[22] Higgins JP Thompson SG. Quantifying heterogeneity in a meta-analysis. Stat Med. (2002) 21:1539–58. 10.1002/sim.1186 12111919

[23] Wathelet M Duhem S Vaiva G Baubet T Habran E Veerapa E. Factors associated with mental health disorders among university students in france confined during the COVID-19 pandemic. JAMA Netw Open. (2020) 3:e2025591. 10.1001/jamanetworkopen.2020.25591 33095252 PMC7584927

[24] Marchini S Zaurino E Bouziotis J Brondino N Delvenne V Delhaye M. Study of resilience and loneliness in youth (18–25 years old) during the COVID-19 pandemic lockdown measures. J Community Psychol. (2020) 49:468–80. 10.1002/jcop.22473 33169377

[25] Busetta G Campolo MG Fiorillo F Pagani L Panarello D Augello V. Effects of COVID-19 lockdown on university students' anxiety disorder in Italy. Genus. (2021) 77:25. 10.1186/s41118-021-00135-5 34658399 PMC8502092

[26] Di Consiglio M Merola S Pascucci T Violani C Couyoumdjian A. The impact of COVID-19 pandemic on italian university students' mental health: changes across the waves. Int J Environ Res Public Health. (2021) 18:9897. 10.3390/ijerph18189897 34574820 PMC8469053

[27] Penteado CT Loureiro JC Pais MV Carvalho CL Sant'Ana LFG Valiengo LCL. Mental health status of psychogeriatric patients during the 2019 new coronavirus disease (COVID-19) pandemic and effects on caregiver burden. Front Psychiatry. (2020) 11:578672. 10.3389/fpsyt.2020.578672 33312138 PMC7704440

[28] Cullen MR Baiocchi M Eggleston K Loftus P Fuchs V. The weaker sex? Vulnerable men and women's resilience to socio-economic disadvantage. SSM Popul Health. (2016) 2:512–24. 10.1016/j.ssmph.2016.06.006 29349167 PMC5757782

[29] Franzoi IG Sauta MD Granieri A. State and trait anxiety among university students: a moderated mediation model of negative affectivity, alexithymia, and housing conditions. Front Psychol. (2020) 11:1255. 10.3389/fpsyg.2020.01255 32587555 PMC7298066

[30] Auger N Lo E Cantinotti M O'Loughlin J. Impulsivity and socio-economic status interact to increase the risk of gambling onset among youth. Addiction. (2010) 105:2176–83. 10.1111/j.1360-0443.2010.03100.x 20840210

[31] Costanza A Rothen S Achab S Thorens G Baertschi M Weber K. Impulsivity and impulsivity-related endophenotypes in suicidal patients with substance use disorders: an exploratory study. Int J Mental Health Addict. (2020) 19:1729–44. 10.1007/s11469-020-00259-3

[32] Frisone F Alibrandi A Settineri S. Problem gambling during Covid-19. MJCP. (2020) 8:3. 10.6092/2282-1619/mjcp-2457

[33] Holmes EA O'Connor RC Perry VH Tracey I Wessely S Arseneault L. Multidisciplinary research priorities for the COVID-19 pandemic: a call for action for mental health science. Lancet Psychiatry. (2020) 7:547–56. 10.1016/S2215-0366(20)30168-1 32304649 PMC7159850

[34] Sampogna G Lovisi GM Zinno F Del Vecchio V Luciano M Gonçalves Loureiro Sol É. Mental health disturbances and related problems in italian university medical students from 2000 to 2020: an integrative review of qualitative and quantitative studies. Medicina. (2020) 57:11. 10.3390/medicina57010011 33374475 PMC7823352

[35] Baertschi M Costanza A Canuto A Weber K. The function of personality in suicidal ideation from the perspective of the interpersonal-psychological theory of suicide. Int J Environ Res Public Health. (2018) 15:636. 10.3390/ijerph15040636 29601506 PMC5923678

[36] Bert F Lo Moro G Corradi A Acampora A Agodi A Brunelli L. Prevalence of depressive symptoms among Italian medical students: the multicentre cross-sectional “PRIMES” study. PLoS ONE. (2020) 15:e0231845. 10.1371/journal.pone.0231845 32302354 PMC7164645

[37] Bert F Ferrara M Boietti E Langiano E Savatteri A Scattaglia M. Depression, suicidal ideation and perceived stress in italian humanities students: a cross-sectional study. Psychol Rep. (2020). 10.1177/0033294120984441 33375898

[38] Piumatti G. Motivation, health-related lifestyles and depression among university students: a longitudinal analysis. Psychiatry Res. (2018) 260:412–7. 10.1016/j.psychres.2017.12.009 29253806

[39] Amerio A Aguglia A Odone A Gianfredi V Serafini G Signorelli C. Covid-19 pandemic impact on mental health of vulnerable populations. Acta Biomed. (2020) 91:95–6. 10.23750/abm.v91i9-S.10112 32701924 PMC8023095

